# Hormonal and laboratory predictors of patent foramen ovale in cryptogenic ischemic events: a SHAP-enhanced logistic regression approach

**DOI:** 10.3389/fneur.2026.1738335

**Published:** 2026-04-17

**Authors:** Yao Zheng, Zhihui Wang, Hongli Pan, Xin Wang, Dan Li, Qi Suo

**Affiliations:** 1 Department of Electrodiagnosis, Jilin Province FAW General Hospital, Changchun, Jilin, China; 2 Department of Clinical Laboratory, Jilin Province FAW General Hospital, Changchun, Jilin, China

**Keywords:** cryptogenic stroke, female, machine learning, patent foramen ovale, SHAP analysis

## Abstract

**Background:**

Patent foramen ovale (PFO) is associated with cryptogenic ischemic events. Detection relies on advanced imaging modalities, yet early detection tools remain limited.

**Methods:**

We developed a predictive model for PFO detection in cryptogenic stroke/TIA patients using clinical and laboratory data. This retrospective study included 300 female patients (18–65 years) with cryptogenic ischemic events (TOAST criteria) who underwent contrast-enhanced transcranial Doppler and echocardiography for PFO assessment. Forty-three variables were analyzed, with LASSO and mRMR feature selection identifying five key predictors: age, estradiol, follicle-stimulating hormone (FSH), D-dimer, and LDL cholesterol. Nine machine learning models were evaluated, with logistic regression selected as the final model. Performance was assessed via AUC, accuracy, sensitivity, specificity, F1 score, and decision curve analysis. SHAP analysis explained feature contributions. We also conducted a prespecified subgroup analysis by reproductive stage (reproductive-age 22–45 years vs. menopausal 45–65 years) to account for stage-related hormonal differences.

**Results:**

Among 300 female patients, 60 (20%) were PFO-positive. PFO carriers were younger (46.8 vs. 53.9 years, *p* < 0.001) with higher estradiol (94.1 vs. 60.3 pg./mL, *p* < 0.001) and D-dimer (2.1 vs. 1.2 mg/L, *p* < 0.001). The logistic regression model achieved an AUC of 0.990, accuracy of 95.6%, sensitivity of 89.5%, and specificity of 97.2%. SHAP analysis highlighted estradiol, D-dimer, and age as top predictors. Findings were consistent across reproductive-stage strata: estradiol and FSH remained differentiating markers (all *p* < 0.05), and the model maintained strong discrimination in both groups (AUC 0.988 in reproductive-age; 0.982 in menopausal).

**Conclusion:**

An interpretable, high-performing model can aid early PFO risk stratification in females; performance was robust across reproductive-age and menopausal strata, and external validation especially in larger female cohorts is warranted. Our model does not assess causality but aims to help clinicians identify those who are most likely to have a PFO, which can then be further investigated to determine the causal role of PFO in the ischemic event.

## 1 Introduction

Ischemic stroke remains a major cause of disability and death worldwide, with over 12 million new cases and 6.5 million stroke-related deaths reported annually (1, 2). While large-artery atherosclerosis, small-vessel disease, and cardioembolism account for most ischemic strokes, a substantial minority remain classified as cryptogenic (stroke of undetermined etiology) (3), particularly among younger individuals lacking conventional vascular risk factors. Mounting evidence suggests that patent foramen ovale (PFO) a remnant of the fetal circulation that persists in approximately 25% of the general adult population plays a causative role in a substantial subset of these events in both males and females (4). Large-scale clinical trials have demonstrated that percutaneous closure of PFO in carefully selected patients can significantly reduce the risk of recurrent stroke (4, 5), underscoring the clinical importance of timely detection. However, the identification of PFO in routine practice remains suboptimal, especially during the acute phase, when diagnostic imaging may be delayed or unavailable (6).

Current diagnostic strategies for PFO rely primarily on contrast-enhanced transesophageal echocardiography (TEE) or transcranial Doppler (TCD), often supplemented by right-heart contrast studies (7). While these modalities remain the gold standard, they are resource-intensive, operator-dependent, and frequently deferred during acute stroke workup particularly in non-tertiary or resource-limited settings (8). Moreover, patient discomfort, procedural contraindications, and variability in technique further complicate access and reliability (9). As a result, many cryptogenic stroke patients remain undiagnosed or experience delays in appropriate secondary prevention (10).

Although clinical scores such as the Risk of Paradoxical Embolism (RoPE) score have been proposed to estimate the probability that a detected PFO is causally related to a cryptogenic stroke (the “PFO-attributable fraction”), rather than the likelihood of PFO presence (11), because these scores were primarily derived from retrospective data, their performance may vary across populations and clinical presentations, and modified RoPE approaches have been proposed to improve risk stratification (12). Importantly, these tools do not incorporate individual biological markers or patient-level pathophysiological profiles, limiting their predictive capacity in personalized medicine contexts (13).

Emerging evidence suggests that circulating biomarkers particularly those related to hormonal status, coagulation activity, and endothelial function may offer mechanistic insight into PFO-associated stroke (8). While PFO-related stroke mechanisms are relevant to both sexes, hormonal milieu may be especially informative in women, and reproductive-stage variation could influence thrombotic propensity and vascular function. Estradiol, for example, modulates endothelial permeability and venous thromboembolism risk, potentially predisposing females to paradoxical embolism in the setting of a right-to-left shunt (14). Follicle-stimulating hormone (FSH) and other female sex-related hormones have also been implicated in vascular reactivity and hemodynamic regulation (15), yet their role in PFO pathogenesis and PFO-associated ischemic events in women remains poorly understood. Additionally, biomarkers such as D-dimer, a marker of fibrinolytic activation, and low-density lipoprotein cholesterol (LDL-C), a surrogate of endothelial dysfunction, may serve as indirect indicators of hypercoagulable states that potentiate embolic risk through a PFO conduit (16). Despite their availability in routine clinical panels, these parameters have not been systematically integrated into predictive frameworks for PFO identification, nor have they been evaluated using interpretable machine learning approaches capable of revealing individualized risk patterns.

Given these gaps, there is a compelling need for a clinically interpretable, data-driven tool to support early identification of PFO among patients with cryptogenic ischemic events. In this study, we focused on a female cohort to test whether routinely available hormonal and laboratory variables improve PFO detection and to explore model behavior across reproductive-stage strata; this sex-specific design does not imply that PFO diagnosis, secondary prevention, or percutaneous closure are applicable only to females. Prior studies have largely overlooked the predictive value of routine biochemical and hormonal variables, and few have adopted robust machine learning pipelines that combine feature selection, model optimization, and transparent interpretability (17).

This approach aims to provide a scalable, transparent, and clinically applicable solution for improving PFO risk stratification in the setting of cryptogenic stroke or TIA, with planned applicability exclusively to females. Accordingly, our conclusions are limited to females and should not be interpreted as restricting PFO diagnostic or treatment principles to one sex.

## 2 Methods

### 2.1 Study design, setting and primary objective

This retrospective, single-center observational study was conducted at a hospital in China. It enrolled female patients admitted to the Department of Neurology between January 2022 and December 2024. The study population consisted females with cryptogenic ischemic stroke or transient ischemic attack (TIA). All participants underwent standardized diagnostic testing for PFO, including contrast-enhanced TCD combined with right-heart contrast echocardiography. The primary aim of this study was to develop and validate a predictive model for PFO detection using routinely available clinical, biochemical, and hormonal indicators. The primary aim of the study was to develop a machine learning model to prioritize PFO testing in individuals who have already been referred for evaluation due to cryptogenic ischemic events. This model identifies individuals with a high likelihood of having a PFO, which can then be confirmed through diagnostic imaging (contrast-enhanced TCD or right-heart contrast echocardiography). It is important to distinguish this from existing scores like the RoPE and PASCAL, which are designed to assess the causal relationship between PFO and ischemic events. Table 1 provides a comparison between our model and existing scores, highlighting the differences in their purpose, focus, and main applications. Our model does not assess causality but rather helps clinicians identify those who are most likely to have a PFO, which can then be further investigated using tools such as RoPE or PASCAL to determine the causal role of PFO in the ischemic event. The current study was approved by the Ethics Committee of the Jilin First Automobile General Hospital (approval number JFH202503064). Due to the retrospective design and anonymized data, written informed consent was waived by the Ethics Committee.

**Table 1:** Comparison of our model with existing scores (RoPE and PASCAL).

Model/Score	Purpose	Focus	Main application
Our model	Prioritize PFO testing in females with ischemic events	PFO detection (without assessing causality)	Identify females who may have PFO and benefit from further diagnostic tests
RoPE	Estimate the risk of paradoxical embolism (PFO causality)	Causality between PFO and ischemic event	Assess whether PFO is likely contributing to the ischemic event
PASCAL	Classify PFO as a causal or non-causal factor	Causality assessment of PFO	Assess causality by anatomical and clinical features of PFO

### 2.2 Study population and eligibility criteria

A total of 300 female patients with cryptogenic ischemic events were retrospectively enrolled in this study. All subjects were consecutively identified from the hospital’s stroke registry database. The inclusion criteria were as follows: (1) age between 18 and 65 years; (2) diagnosis of cryptogenic ischemic stroke or TIA according to the TOAST classification system, with no identifiable etiology following a comprehensive standard workup; and (3) completion of PFO assessment using contrast-enhanced TCD combined with right-heart contrast echocardiography. Both cryptogenic ischemic stroke and cryptogenic TIA were considered index events, and no additional TIA events outside of the index event were included. Exclusion criteria included: (1) evidence of a determined stroke etiology, such as large artery atherosclerosis, small vessel occlusion, or major cardioembolic sources (e.g., atrial fibrillation); (2) incomplete clinical, laboratory, or imaging data; and (3) a history of prior PFO closure or cardiac surgery.; and (4) known active malignancy at the time of admission or during the index hospitalization (excluded by medical history and chart review).

Stroke versus TIA was diagnosed by vascular neurologists based on clinical presentation and neuroimaging. Ischemic stroke was defined as a focal neurological deficit with imaging evidence of acute infarction, preferentially confirmed by brain MRI with diffusion-weighted imaging (DWI) when available. TIA was defined as transient focal neurological symptoms without evidence of acute infarction on neuroimaging (MRI-DWI or, when MRI was not available/feasible, CT and clinical assessment). To classify events as cryptogenic and exclude alternative etiologies, all patients underwent a standardized etiologic workup consistent with TOAST criteria, including brain CT and/or MRI, intracranial and extracranial vascular imaging (e.g., CTA/MRA and/or carotid ultrasound), and cardiac evaluation (e.g., 12-lead ECG, inpatient telemetry and/or 24-h Holter monitoring, and transthoracic echocardiography (TTE)) to exclude large-artery atherosclerosis, small-vessel occlusion, and major cardioembolic sources (including atrial fibrillation). Additional laboratory testing was performed as clinically indicated.

PFO status was determined based on the combined imaging findings. A right-to-left shunt observed at rest or during a Valsalva maneuver was considered indicative of PFO. In patients evaluated with the dual-modality approach, PFO was diagnosed when microbubbles were visualized in the middle cerebral artery signal on contrast-enhanced TCD and/or in the left atrium on contrast-enhanced TTE within 25 s of contrast injection. Of these 300 female patients, 60 were PFO-positive and 240 were PFO-negative. Patients were randomly assigned to training (70%) and test (30%) sets. The distribution of PFO-positive and PFO-negative cases was consistent between the two sets (training set: 20% vs. 80%; test set: 20% vs. 80%), ensuring no significant imbalance. The study cohort included adult females aged 18–65 years. For analyses, participants were grouped as reproductive-age (22–45 years) or postmenopausal (51–65 years). These subgroups were used for subgroup analysis to assess the predictive performance of the model across different stages of reproductive health. Additionally, patients were referred for PFO evaluation based on clinical criteria, including symptoms suggestive of cryptogenic ischemic events, history of stroke/TIA, and other clinical indications that warrant further assessment for PFO Referral was guided by standard medical practice and clinical judgment as per the institutional guidelines. PFO referral was based on the presence of cryptogenic ischemic events, including TIA and stroke, in patients with no other identifiable vascular causes. Patients meeting these criteria were referred for PFO evaluation using contrast-enhanced transcranial Doppler (TCD) or contrast-enhanced TTE.

### 2.3 Data collection and candidate variables

Data were extracted retrospectively from the hospital’s electronic medical records system by two trained researchers using a standardized data collection template. All information was cross-validated against discharge summaries and laboratory reports to ensure consistency and completeness. Blood samples were collected within 48 h of admission; exact symptom-onset–to–blood-draw times were not consistently available in this retrospective dataset, so a median (IQR) could not be calculated. A total of 43 candidate variables were collected and grouped into the following four domains: (1) Demographic and clinical characteristics: age (years), body mass index (BMI, kg/m^2^), smoking history (yes/no), pregnancy history (yes/no), menopausal status (premenopausal/postmenopausal), ischemic event type (stroke or TIA), lesion location (categorized as cortical, deep, brainstem, or cerebellar), and whether the event was a first-ever occurrence. (2) Hormonal indicators: serum estradiol (E2, pg./mL), progesterone (P, ng/mL), prolactin (PRL, ng/mL), follicle-stimulating hormone (FSH, IU/L), luteinizing hormone (LH, IU/L), and testosterone (T, ng/dL), all measured by chemiluminescent immunoassays within 48 h of admission; analyses were specific to females. (3) Coagulation and cardiac biomarkers: D-dimer (mg/L), fibrinogen (FIB, g/L), prothrombin time (PT, s), activated partial thromboplastin time (APTT, s), platelet count (PLT, ×10^9^/L), high-sensitivity cardiac troponin I (hs-cTnI, pg./mL), and N-terminal pro-brain natriuretic peptide (NT-proBNP, pg./mL). (4) Routine laboratory parameters: blood urea nitrogen (BUN, mmol/L), serum creatinine (Scr, μmol/L), total cholesterol (TC, mmol/L), triglycerides (TG, mmol/L), high-density lipoprotein cholesterol (HDL-C, mmol/L), low-density lipoprotein cholesterol (LDL-C, mmol/L), fasting plasma glucose (FPG, mmol/L), glycated hemoglobin (HbA1c, %), white blood cell count (WBC, ×10^9^/L), and neutrophil percentage (NEU%). All laboratory assays were performed in the hospital’s central laboratory using certified equipment, following standardized operating procedures. Variable selection and preprocessing steps are described in Section 2.5.

Across the 43 candidate variables, the overall proportion of missing values was low, with no variable exceeding 5% missingness. The variables with the highest missing rates were hormonal indicators (estradiol, progesterone, FSH, and LH), each missing in approximately 3–5% of cases, largely due to incomplete laboratory sampling at admission. All other variables had <2% missing values. Details of missingness for each variable are provided in Supplementary Table S1.

### 2.4 Outcome definition and grouping strategy

The primary outcome of interest was the presence or absence of a PFO, operationalized as a binary classification variable (PFO-positive vs. PFO-negative). PFO status was assessed using contrast-enhanced TCD combined with contrast-enhanced TTE, procedures routinely performed by experienced sonographers in the Department of Ultrasound. Examinations adhered to standardized protocols involving the administration of agitated saline contrast, both at rest and during a Valsalva maneuver. A positive result was defined as the appearance of one or more microbubble signals within the middle cerebral artery on TCD and/or within the left atrium on contrast-enhanced TTE within 25 s following contrast injection.

For the purpose of model development, the entire study cohort (*n* = 300 female patients) was categorized into a PFO-positive group (*n* = 60; 20%) and a PFO-negative group (*n* = 240; 80%) based on diagnostic findings. Participants were then randomly allocated into a training set (70%, *n* = 210) and a test set (30%, *n* = 90) using stratified sampling to preserve the original distribution of the outcome variable. Dataset split and evaluation strategy. The full cohort (*n* = 300) was divided once using stratified random sampling into a training set (70%, *n* = 210) and a held-out test set (30%, *n* = 90), preserving the proportion of PFO-positive and PFO-negative cases. All model development steps including preprocessing, feature selection, and hyperparameter tuning were performed exclusively within the training set. Model selection and internal performance estimation were conducted using stratified 10-fold cross-validation within the training set, and bootstrapping within the training set was used to assess optimism. The held-out test set was not used for feature selection, tuning, or model selection and was used only once for final performance evaluation; thus, it represents an internal held-out test rather than external validation. Throughout this manuscript, we use the terms training set and held-out test set and avoid the ambiguous term “validation set.” The training set was employed for feature selection, model building, and internal validation using 10-fold cross-validation. The test set, derived solely from the same cohort, serves as a held-out test set and does not constitute external validation. The held-out test set was subsequently used to assess the predictive performance of the finalized model.

For subgroup analysis, participants were categorized by reproductive stage as reproductive-age (22–45 years) and menopausal (45–65 years) to account for physiological hormonal differences; primary model development used the overall cohort, with subgroup comparisons to assess consistency of predictors and discrimination.

### 2.5 Causal likelihood scoring

In addition to the primary biomarker-based model, we assessed the probability that a detected PFO was causally related to the index ischemic event using two established classification systems: the Risk of Paradoxical Embolism (RoPE) score and the PASCAL classification. The RoPE score was calculated for all female patients based on age, presence of cortical infarct, and absence of traditional vascular risk factors, as described by Kent et al. (18). Scores were categorized into low (0–3), intermediate (4–6), and high (7–10) likelihood of PFO-related stroke. PASCAL classification was applied only when shunt grade and atrial septal aneurysm data were available and only among patients with confirmed PFO, consistent with its intended use.

### 2.6 Model development and evaluation

Model development followed a structured machine learning pipeline consisting of variable selection, multi-model classification, final model construction, and comprehensive performance evaluation. All analyses were conducted in R version 4.2.3 and Python version 3.9.13. Key packages included glmnet (version 4.1–7), sklearn (version 0.24.2), xgboost (version 1.5.1), lightgbm (version 3.3.2), and shap (version 0.41.0).

Preprocessing steps: Continuous variables were standardized to zero mean and unit variance. To improve normality and model stability, log-transformation was applied to skewed distributions, including estradiol, FSH, and D-dimer. Missing data were minimal (<5% for any variable) and were imputed using multiple imputation by chained equations (MICE), assuming data were missing at random. For sensitivity analysis, complete-case analyses were also performed, yielding consistent results.

### 2.7 Feature selection

A two-step feature selection process was employed to identify the most informative predictors. Initially, least absolute shrinkage and selection operator (LASSO) logistic regression with 10-fold cross-validation was implemented using the glmnet package in R. The optimal regularization parameter (*λ*) was determined based on the value that minimized the mean squared error, resulting in the selection of 24 variables with non-zero coefficients. To further reduce redundancy and enhance model interpretability, the mRMR method was applied to the LASSO-derived features using the mRMRe package in R (version 2.1.0). This process yielded five final predictors for subsequent modeling: age, estradiol (E2), FSH, D-dimer, and LDL-C. To improve transparency, we report the complete mRMR output (selection order and scores) for all LASSO-retained variables in Supplementary Table S2; the final five predictors correspond to the top-ranked features by mRMR, reflecting high relevance to PFO status with minimal redundancy.

#### 2.7.1 Model construction

Using the five selected predictors, nine classification models were developed and compared: logistic regression, eXtreme Gradient Boosting (XGBoost), Light Gradient Boosting Machine (LightGBM), Random Forest, Adaptive Boosting (AdaBoost), Gradient Boosting Decision Tree (GBDT), Decision Tree, Gaussian Naïve Bayes (GNB), and Complement Naïve Bayes (CNB). Model training was conducted on the training dataset (*n* = 210) using stratified 10-fold cross-validation to preserve the class distribution. Hyperparameter tuning was performed via grid search within the training folds, and the hyperparameters explored included: regularization strength (*λ*) for logistic regression, learning rate (*η*) for XGBoost, number of leaves for LightGBM, and number of trees (n_estimators) for Random Forest, with the ranges specified in the manuscript. The independent test set (*n* = 90), which was not involved in the training process, was reserved for evaluating final model performance.

Model selection justification: Although ensemble methods such as XGBoost, LightGBM, and Random Forest achieved near-perfect AUCs in the training set, they showed evidence of overfitting and slightly reduced calibration in the held-out test set. Logistic regression, by contrast, demonstrated consistently high AUCs across training, validation, and independent testing, with superior calibration and generalizability. Moreover, logistic regression offers clinical interpretability, which was further enhanced through SHAP analysis, enabling both global and individual-level explanations. Given the importance of transparency and reproducibility in clinical decision-making, logistic regression was chosen as the final deployment model despite marginally higher AUCs observed in some ensemble models.

#### 2.7.2 Model evaluation

Model discrimination was primarily assessed using the area under the receiver operating characteristic (ROC) curve (AUC). Additional performance metrics included classification accuracy, sensitivity, specificity, precision, negative predictive value (NPV), F1 score, and the Kolmogorov–Smirnov (KS) statistic. Calibration performance was evaluated using calibration plots, and clinical utility was assessed through decision curve analysis (DCA), implemented using the rmda package in R (version 1.6). Precision–recall (PR) curves and the area under the PR curve (AP) were generated to address class imbalance sensitivity. Comparative analysis of model performance was conducted using pairwise AUC comparisons via the DeLong test.

The final logistic regression model was selected based on its robust performance, interpretability, and favorable balance between discrimination and calibration. Its performance was further explained using SHAP, described in Section2.7.3.

#### 2.7.3 SHAP-based interpretation

We applied SHapley Additive exPlanations (SHAP) to the final logistic regression model to quantify each predictor’s contribution to the predicted probability of PFO. Although logistic regression (LR) is intrinsically interpretable through its regression coefficients, we additionally used SHAP to provide complementary interpretability at both the global and individual level. LR coefficients quantify the direction and magnitude of association on the log-odds scale (and can be expressed as odds ratios), whereas SHAP values quantify each predictor’s contribution to an individual patient’s predicted probability relative to a baseline, facilitating case-level explanations and consistent comparison of feature contributions on the prediction scale. SHAP values were computed using the final multivariable LR model fitted with the five selected predictors (age, estradiol, FSH, D-dimer, and LDL-C). Global feature importance was summarized using mean absolute SHAP values, and individual predictions were illustrated using SHAP-based force/waterfall plots. To improve transparency and enable direct comparison between coefficient-based interpretation and SHAP-based importance, we additionally report the complete multivariable LR coefficients (*β*, standard error, odds ratio, 95% confidence interval, and *p*-value) for the final five predictors. Implementation details (package versions, plotting procedures) are provided in the Supplementary Figure S1.

### 2.8 Statistical analysis

All analyses were conducted using R software (version 4.2.2) and Python (version 3.9.13). Continuous variables were expressed as mean ± standard deviation (SD) or median (interquartile range, IQR) depending on distribution, and compared using Student’s *t*-test or Mann–Whitney U test. Categorical variables were presented as counts and percentages, and compared using chi-square or Fisher’s exact test. Logistic regression analyses were adjusted for age and other covariates as specified in each model; because the cohort included females only, sex was not included as a covariate. For continuous variables, we assessed adjusted odds ratios (ORs) with 95% confidence intervals (CIs) and corresponding *p*-values.

Shapley additive explanation (SHAP) values were computed for the final logistic regression model to interpret feature contributions at both the global and individual-patient level. Statistical significance was set at a two-tailed *p*-value < 0.05.

## 3 Results

### 3.1 Distinct clinical and hormonal profiles in patients with and without PFO

A total of 300 adult female patients with cryptogenic ischemic events were included in the analysis. Among them, 60 (20%) were diagnosed with PFO, while 240 (80%) were classified as PFO-negative based on results from contrast-enhanced TCD combined with right-heart contrast echocardiography. Compared with the non-PFO group, female patients in the PFO group were significantly younger (46.8 ± 7.0 vs. 53.9 ± 5.4 years; *p* < 0.001) and had lower BMI values (22.8 ± 2.3 vs. 23.6 ± 2.6 kg/m^2^; *p* = 0.047). The incidence of TIA was notably higher among PFO-positive females (22% vs. 11%; *p* = 0.028), and cortical lesions were more frequently detected (50% vs. 24%; *p* = 0.034) (Table 2). Among index events, cryptogenic TIA was more frequent in the PFO-positive female group (22%) compared with the PFO-negative female group (11%; *p* = 0.028). In this study, TIAs were counted as index cryptogenic ischemic events according to TOAST classification criteria, and not as additional events. In terms of laboratory parameters, PFO-positive females had significantly higher serum estradiol levels (94.1 ± 25.7 vs. 60.3 ± 19.4 pg./mL; *p* < 0.001) and D-dimer concentrations (2.1 ± 0.8 vs. 1.2 ± 0.7 mg/L; *p* < 0.001). Progesterone levels were also modestly elevated (6.0 ± 1.8 vs. 5.3 ± 1.7 ng/mL; *p* = 0.032). No significant differences were observed for prolactin or other hormonal markers. Additionally, serum creatinine was lower in PFO-positive female group (64.7 ± 10.2 vs. 69.3 ± 11.8 μmol/L; *p* = 0.018), while HDL-C levels were slightly higher (1.5 ± 0.3 vs. 1.4 ± 0.3 mmol/L; *p* = 0.045). In an age-adjusted exploratory logistic regression model, estradiol (adjusted OR = 2.85, 95% CI 1.95–4.16; *p* < 0.001) and FSH (adjusted OR = 1.62, 95% CI 1.10–2.39; *p* = 0.015) remained associated with PFO status. Because FSH is correlated with reproductive hormonal milieu, particularly estradiol and with age, its estimated association can differ between an age-adjusted model and the fully adjusted multivariable model; thus, the final multivariable estimates in Table 3 should be interpreted as conditional effects after accounting for correlated predictors and covariates.

**Table 2:** Baseline characteristics of female patients with cryptogenic ischemic events stratified by PFO status (*N* = 300).

Variable	PFO positive (*n* = 60)	PFO negative (*n* = 240)	Test statistic	*p* value	*β* (final logistic regression model^a^)	Importance rank (mean |SHAP|^a^)
Age, years	46.8 ± 7.0	53.9 ± 5.4	*t* = −7.12	<0.001	*β* = −0.42	Rank = 3
BMI, kg/m^2^	22.8 ± 2.3	23.6 ± 2.6	*t* = −2.01	0.047	–	–
Smoking history, *n* (%)	11 (18%)	56 (23%)	χ^2^ = 0.67	0.412	–	–
Pregnancy history, *n* (%)	52 (87%)	199 (83%)	χ^2^ = 0.52	0.474	–	–
Postmenopausal status, *n* (%)	40 (67%)	171 (71%)	χ^2^ = 0.30	0.586	–	–
Ischemic event type, *n* (%)			χ^2^ = 4.84	0.028	–	–
Stroke	47 (78%)	214 (89%)			–	–
TIA	13 (22%)	26 (11%)			–	–
Lesion location, *n* (%)			χ^2^ = 8.73	0.034	–	–
Cortical	30 (50%)	57 (24%)			–	–
Subcortical	18 (30%)	77 (32%)			–	–
Brainstem	8 (13%)	59 (25%)			–	–
Cerebellum	4 (7%)	20 (8%)			–	–
Estradiol (E2), pg./mL	94.1 ± 25.7	60.3 ± 19.4	*t* = 9.47	<0.001	*β* = +0.36	Rank = 1
Progesterone (P), ng/mL	6.0 ± 1.8	5.3 ± 1.7	*t* = 2.18	0.032	–	–
Prolactin (PRL), ng/mL	13.9 ± 5.1	13.5 ± 4.8	*t* = 0.64	0.526	–	–
FSH, IU/L	14.8 ± 5.3	16.1 ± 5.8	*t* = −1.70	0.092	*β* = −0.21	Rank = 5
LH, IU/L	9.1 ± 3.6	9.4 ± 3.9	*t* = −0.76	0.448	–	–
Testosterone (T), ng/dL	32.5 ± 6.8	30.3 ± 7.1	*t* = 1.89	0.061	–	–
D-dimer, mg/L	2.1 ± 0.8	1.2 ± 0.7	*t* = 8.22	<0.001	*β* = +0.41	Rank = 2
Fibrinogen (FIB), g/L	3.1 ± 0.5	3.2 ± 0.6	*t* = −1.26	0.210	–	–
PT, s	12.6 ± 0.6	12.7 ± 0.6	*t* = −0.91	0.367	–	–
APTT, s	31.1 ± 3.2	30.5 ± 3.1	*t* = 1.69	0.094	–	–
Platelet count, ×10^9^/L	232 ± 52	240 ± 49	*t* = −1.34	0.182	–	–
hs-cTnI, pg./mL	5.5 ± 1.7	5.8 ± 1.9	*t* = −1.05	0.293	–	–
NT-proBNP, pg./mL	91 ± 26	95 ± 28	*t* = −1.12	0.267	–	–
BUN, mmol/L	4.5 ± 1.0	4.7 ± 1.1	*t* = −1.30	0.193	–	–
Serum creatinine, μmol/L	64.7 ± 10.2	69.3 ± 11.8	*t* = −2.44	0.018	–	–
Total cholesterol (TC), mmol/L	4.8 ± 0.6	4.9 ± 0.6	*t* = −0.84	0.402	–	–
Triglycerides (TG), mmol/L	1.3 ± 0.4	1.4 ± 0.4	*t* = −1.35	0.176	–	–
HDL-C, mmol/L	1.5 ± 0.3	1.4 ± 0.3	*t* = 2.01	0.045	–	–
LDL-C, mmol/L	2.7 ± 0.5	2.9 ± 0.5	*t* = −1.97	0.049	*β* = −0.27	Rank = 4
Fasting glucose, mmol/L	5.2 ± 0.6	5.3 ± 0.6	*t* = −1.23	0.224	–	–
HbA1c, %	5.7 ± 0.4	5.8 ± 0.5	*t* = −1.29	0.196	–	–
White blood cell count, ×10^9^/L	6.3 ± 1.2	6.5 ± 1.3	*t* = −1.23	0.243	–	–
Neutrophil percentage, %	58.1 ± 7.9	59.3 ± 8.1	*t* = −1.15	0.251	–	–

a *β* coefficients are from the final logistic regression prediction model for PFO presence using five predictors: age, estradiol (E2), FSH, D-dimer, and LDL-C. “Importance rank” reflects mean absolute SHAP values derived from the same final model (higher rank indicates greater contribution to predictions). For variables not included in the final model, *β* and SHAP rank are not applicable and are shown as “–.”

**Table 3:** Final predictors: mRMR selection order, multivariable LR coefficients, and SHAP importance.

Predictor (final 5)	mRMR selection order	LR *β* (log-odds)	OR = exp.(*β*)	95% CI for OR	SHAP rank (mean |SHAP|)
Estradiol (E2)	1	0.36	1.43	(1.23–1.66)	1
FSH	2	−0.21	0.81	(0.70–0.94)	5
D-dimer	3	0.41	1.51	(1.24–1.83)	2
Age (years)	4	−0.42	0.66	(0.54–0.79)	3
LDL-C	5	−0.27	0.76	(0.61–0.95)	4

In analyses stratified by reproductive stage, estradiol remained significantly higher and FSH significantly lower in PFO-positive and PFO-negative females in both the reproductive-age (22–45 years) and menopausal (45–65 years) strata (all *p* < 0.05). The final logistic regression prediction model included age, estradiol (E2), FSH, D-dimer, and LDL-C, and it maintained excellent discrimination within each stratum (AUC 0.988 reproductive-age; 0.982 menopausal), supporting model stability across reproductive stages.

### 3.2 Identification of key predictors via LASSO and mRMR feature selection

**Figure 1:**
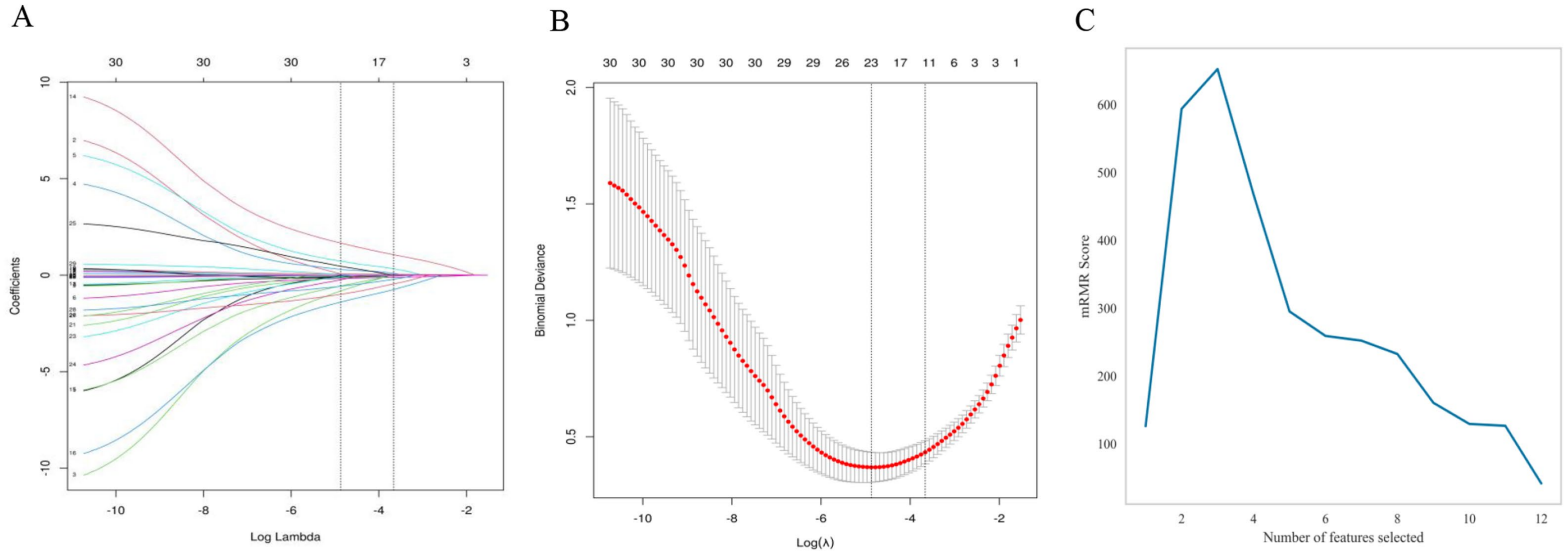
Feature selection using LASSO regression and mRMR analysis. **(A)** LASSO coefficient profiles of 30 baseline variables. Each colored curve represents the trajectory of a variable’s coefficient as the regularization parameter (*λ*) changes. The vertical dashed lines indicate the optimal λ values identified by 10-fold cross-validation. **(B)** Cross-validation plot of binomial deviance against log(λ). The left dotted line represents the λ value that minimizes the mean cross-validated error, and the right dotted line marks the most parsimonious model within one standard error. **(C)** mRMR feature ranking showing the trade-off between relevance and redundancy. The five top-ranked variables estradiol (E2), follicle-stimulating hormone (FSH), D-dimer, age, and LDL-C were selected for further modeling.

To identify the most informative variables for predicting PFO status, we initially applied LASSO logistic regression with 10-fold cross-validation using a binomial model. As illustrated in the coefficient path diagram and cross-validation curve (Figures 1A,B), the optimal penalty parameter (*λ* = 0.008) yielded a model comprising 24 variables with non-zero coefficients. These included demographics, clinical, hormonal, and laboratory variables such as smoking history, ischemic event type (TIA), lesion location (cortical), age, BMI, estradiol (E2), FSH, testosterone, D-dimer, fibrinogen, PT, platelet count, hs-cTnI, BUN, total cholesterol, triglycerides, HDL-C, LDL-C, HbA1c, white blood cell count, and neutrophil percentage. The full list of selected variables and coefficients is provided in Supplementary Table S3. To further refine feature selection by minimizing redundancy and enhancing interpretability, we employed the mRMR algorithm on the LASSO-derived features. This process identified five key predictors with the highest discriminative utility: estradiol (E2), FSH, D-dimer, age, and LDL-C (Figure 1C). These variables were retained for downstream multi-model classification and logistic regression modeling.

To improve interpretability and transparency, we report the complete multivariable logistic regression results for the final model containing these five predictors (age, estradiol, FSH, D-dimer, and LDL-C). Table 3 summarizes the regression coefficients (*β*), standard errors, odds ratios (ORs) with 95% confidence intervals, and *p*-values for each predictor. In the same table, we present the mRMR selection order and SHAP global importance (mean absolute SHAP values and rank) to enable direct comparison between coefficient-based interpretation and SHAP-based feature contributions. Overall, the SHAP-derived global importance ranking was largely concordant with the multivariable LR effect sizes, supporting consistent interpretation between these two approaches.

### 3.3 Comparative performance of nine machine learning models in PFO prediction

**Figure 2:**
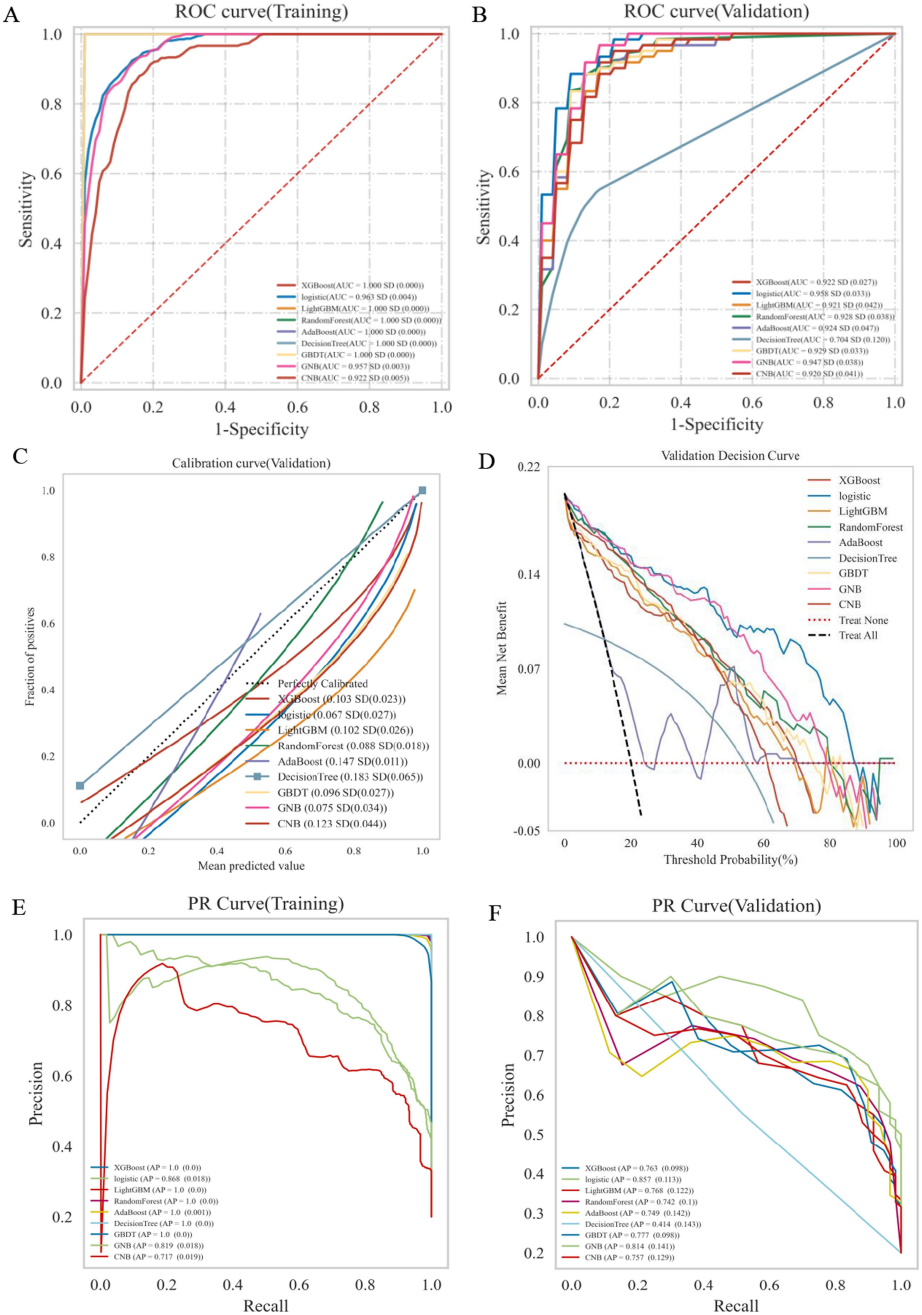
Performance comparison of nine classification models in predicting PFO status. **(A)** ROC curves for the training set. Several ensemble models show perfect AUC values (1.000), indicating potential overfitting, while logistic regression achieves high but more realistic performance (AUC = 0.963). **(B)** ROC curves for the validation set. Logistic regression demonstrates the highest AUC (0.958), outperforming other models. The decision tree model shows the lowest AUC (0.704). **(C)** Calibration curves for the validation set. Logistic regression provides the best agreement between predicted and observed probabilities. **(D)** Decision curve analysis (DCA) in the validation set. Logistic regression yields the highest net clinical benefit across most threshold probabilities. **(E)** Precision-recall (PR) curves for the training set. Logistic regression achieves an average precision of 0.868, while ensemble models show overfitting with a perfect average precision (AP = 1.000). **(F)** PR curves for the validation set. Logistic regression outperforms other models with an average precision of 0.857.

Nine classification algorithms were constructed using five selected predictors (estradiol, FSH, D-dimer, age, and LDL-C) and their performance in discriminating PFO status was evaluated using a 70:30 training–validation split. In the training set (*n* = 210 female patients), ensemble models such as XGBoost, LightGBM, Random Forest, AdaBoost, and GBDT achieved perfect or near-perfect metrics across AUC, sensitivity, and specificity (AUC = 1.000), whereas logistic regression exhibited high but non-saturated performance (AUC = 0.963) (Figure 2A; Supplementary Table S4). In the validation set (*n* = 90), logistic regression demonstrated the highest AUC (0.958), followed closely by GBDT (0.929), Random Forest (0.928), and GNB (0.947), while the Decision Tree model yielded the lowest AUC (0.704) (Figure 2B; Supplementary Table S5). Calibration analysis showed that logistic regression provided the best agreement between predicted probabilities and observed outcomes, followed by GNB (Figure 2C). DCA revealed that logistic regression yielded the greatest net clinical benefit across a range of threshold probabilities (Figure 2D). In the PR assessment, logistic regression achieved the highest average precision (AP = 0.857) in the validation set, outperforming ensemble and naïve Bayes models (Figures 2E,F). Pairwise DeLong tests indicated no statistically significant differences in AUC between logistic regression and other top-performing models, except for significantly lower performance in the Decision Tree and AdaBoost models (Supplementary Tables S4A,B).

### 3.4 Development and validation of a logistic regression model for PFO discrimination

**Figure 3:**
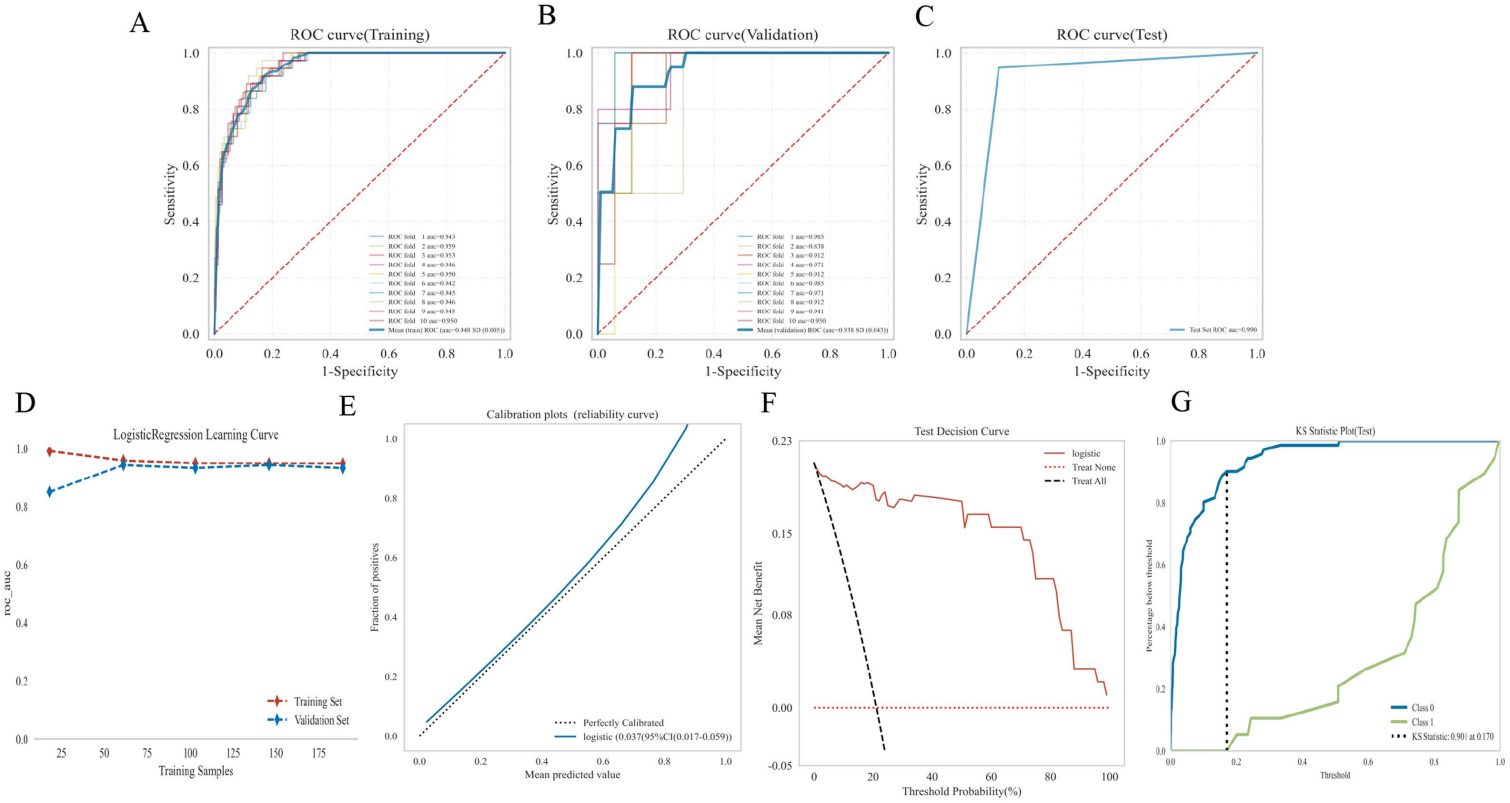
Performance of the final logistic regression model in PFO prediction. **(A)** ROC curve for the 10-fold cross-validation in the training set (*n* = 210), with an AUC of 0.948 (SD = 0.005). **(B)** ROC curve in the held-out test set (*n* = 90), with a mean AUC of 0.938 (SD = 0.043). **(C)** ROC curve in the independent test set (*n* = 90), with an AUC of 0.990, demonstrating high performance. **(D)** Calibration plot in the test set showing good agreement between predicted and observed risk (calibration error = 0.037, 95% CI: 0.017–0.059). **(E)** Learning curve showing stable model performance as sample size increases. **(F)** Decision curve analysis (DCA) in the test set, demonstrating net clinical benefit across threshold probabilities of 5–30%. **(G)** Kolmogorov–Smirnov (KS) plot in the test set, with a KS statistic of 0.901, indicating strong separation between predicted risk distributions in PFO-positive and PFO-negative cases.

A logistic regression model incorporating five predictors (age, estradiol, FSH, D-dimer, and LDL-C) was constructed to classify PFO status. In the training cohort (*n* = 210), 10-fold cross-validation yielded a mean AUC of 0.948 (SD 0.005) (Figure 3A), and cross-validated AUC across folds was 0.938 (SD 0.043) (Figure 3B). When applied to the independent held-out test set (*n* = 90), the model achieved an AUC of 0.990, with accuracy of 0.956, sensitivity of 0.895, specificity of 0.972, and an F1 score of 0.895 (Figure 3C; Supplementary Table S6). Calibration analysis in the held-out test set showed close agreement between predicted and observed probabilities (Figure 3D). The learning curve demonstrated stable performance with increasing sample size (Figure 3E). DCA indicated a net clinical benefit across relevant probability thresholds (Figure 3F), and the KS plot revealed strong separation between predicted risk distributions in PFO-positive and negative female patients (KS statistic = 0.901) (Figure 3G). Detailed performance metrics are provided in Supplementary Table S7. In response to the concern of potential optimism from using a single data split, we performed additional internal validation within the training set, including bootstrapping and repeated cross-validation. The bootstrapping process (1,000 resamples) yielded an average AUC of 0.986 (95% CI: 0.976–0.994), confirming the robustness of the model’s performance across different subsets of the data. Furthermore, repeated 10-fold cross-validation was conducted, and the mean AUC across iterations was 0.984 (SD 0.010), providing further evidence that the model is not overfitted to a single data split. These additional validation procedures indicate that the model’s performance is stable and generalizable across different datasets. Decision curve analysis (DCA) demonstrated that the model provided greater net clinical benefit than either the treat-all or treat-none strategies across threshold probabilities ranging from 10 to 60%. Within this range, a threshold of approximately 30% yielded an optimal balance between sensitivity and specificity, supporting its potential clinical utility for identifying patients at higher likelihood of PFO. This model was subsequently subjected to SHAP interpretation (see Section 3.5).

### 3.5 SHAP-based interpretation reveals feature contributions and individual-level risk patterns

To enhance interpretability, we applied SHAP to the final model. SHAP identified estradiol, D-dimer, and age as the strongest contributors to PFO prediction, consistent with model coefficients. Full methods and all SHAP visualizations (summary, dependence, and patient-level force plots) are provided in the Supplementary Figure S1.

### 3.6 RoPE and PASCAL distributions

Among the female cohort, the median (IQR) RoPE score was 6 (4–8). RoPE categories were: low (0–3) in 68 patients (22.7%), intermediate (4–6) in 124 patients (41.3%), and high (7–10) in 108 patients (36.0%). In the PFO-positive group, 59.3% had a high RoPE score compared with 12.4% in the PFO-negative group.

PASCAL classification was available for a subset of patients with complete anatomic/shunt data; because PASCAL is intended to assess causal-likelihood in confirmed PFO, it is reported only among PFO-positive patients with available data and is not applicable to PFO-negative patients (Table 4).

**Table 4:** Performance of the final biomarker-based model within RoPE categories and applicable PASCAL subset.

Category	*n* (%)	PFO positive *n* (%)	PFO negative *n* (%)	AUC (95% CI) of final model^a^
(A) RoPE score categories (entire cohort; descriptive subgroup AUCs)				
Low (0–3)	68 (22.7)	10 (14.7)	58 (85.3)	0.964 (0.940–0.983)
Intermediate (4–6)	124 (41.3)	50 (40.3)	74 (59.7)	0.972 (0.952–0.988)
High (7–10)	108 (36.0)	64 (59.3)	44 (40.7)	0.987 (0.971–0.995)
(B) PASCAL classification (PFO-positive only; applicable subset)^b^				
Unlikely	NA	12 (among PFO + with PASCAL data)	NA	NA
Possible	NA	39 (among PFO + with PASCAL data)	NA	NA
Probable	NA	66 (among PFO + with PASCAL data)	NA	NA

a AUCs are computed by applying the single final biomarker-based model (age, estradiol, FSH, D-dimer, LDL-C) to each subgroup; no within-stratum model refitting was performed.

b PASCAL classification is intended to assess the causal likelihood of PFO-associated stroke and requires PFO-related anatomical/shunt information; therefore, it is reported only among PFO-positive patients with available data and is not applicable to PFO-negative patients. Accordingly, AUC is not reported for PASCAL categories because discrimination metrics require both PFO-positive and PFO-negative cases.

## 4 Discussion

This study presents a clinically interpretable machine learning model for predicting the presence of PFO in females with cryptogenic ischemic stroke or TIA, using routinely available demographic, hormonal, and biochemical indicators. Among 300 adult female patients, five key predictors age, estradiol, FSH, D-dimer, and LDL-C were identified for accurate PFO stratification. Rather than emphasizing a single “most powerful” predictor, we observed that estradiol, D-dimer, and age were consistently among the leading contributors across complementary analyses (mRMR selection, multivariable logistic regression coefficients/odds ratios, and SHAP-based feature contributions). We therefore interpret predictor importance based on convergence across methods, recognizing that rankings can vary depending on the metric and scale (e.g., coefficient magnitude on the log-odds scale versus contribution to predicted probability).

To place these findings within current clinical frameworks, we additionally summarized RoPE and PASCAL scores, which are commonly used to estimate the causal likelihood that an identified PFO is pathogenic in cryptogenic events.

The findings extend beyond previous clinical scoring tools by integrating female-specific hormonal profiles and coagulation biomarkers, thereby offering a biologically grounded, data-driven approach to risk stratification in a population where early diagnosis of PFO is often delayed or missed (19). All assays and analyses were restricted to females, and multivariable models were adjusted for age only; descriptive statistics are presented for the female cohort (Table 2). The model’s performance and transparency suggest it may serve as a valuable adjunct to clinical decision-making, particularly in settings where immediate access to contrast-enhanced echocardiography or TCD is limited. Subgroup analyses demonstrated that predictive performance and key hormonal associations were robust across reproductive-age and menopausal strata. This supports the biological plausibility and generalizability of the model within a female -only cohort (20).

Building on these findings, our study provides several insights into the potential mechanisms linking hormonal and coagulation profiles to PFO presence among patients presenting with cryptogenic ischemic stroke/TIA. The Estradiol showed a consistent association with PFO status and ranked among the top contributors across methods, aligning with prior evidence implicating estrogenic activity in the modulation of vascular tone, endothelial permeability, and venous thromboembolism risk. Estradiol may enhance coagulability through both genomic and non-genomic pathways, increasing thrombin generation and reducing fibrinolytic activity (21). In the presence of a functional right-to-left shunt, such changes could facilitate paradoxical embolism, particularly in females with subclinical venous thrombi. The association between higher D-dimer levels and PFO presence further supports a thrombosis-prone state as a key component of this embolic pathway. While D-dimer is traditionally used in venous thromboembolism diagnostics, its elevation in PFO-positive females may reflect ongoing fibrin turnover linked to occult venous clot formation or microembolization. Our results suggest that the interplay between hormonal status and coagulation activity could modulate the clinical significance of PFO, distinguishing it as an active embolic conduit rather than an incidental anatomical variant. This hypothesis is reinforced by the selection of FSH as an independent predictor an often-overlooked marker in stroke risk profiling (22). As FSH levels increase with reproductive aging, they may serve as an indirect surrogate for broader endocrine transitions that influence vascular dynamics, including shifts in sympathetic tone and endothelial responsiveness. Importantly, because our study design focused on identifying PFO among patients with cryptogenic stroke/TIA, these findings should not be interpreted as predicting which individuals with known PFO will develop stroke/TIA. Prospective studies in cohorts with confirmed PFO are needed to determine whether these biomarkers are associated with future ischemic events or recurrence risk. Estradiol and FSH were available for the majority of participants, with low missingness (approximately 3–5%); missing values were handled using the imputation strategy described in the Methods.

However, we acknowledge that the biomarkers measured in this study estradiol, FSH, and D-dimer were collected within 48 h of the ischemic event. This timing may introduce confounding variables, as biomarker levels can be influenced by acute-phase responses, stress, inflammation, and treatments like anticoagulation therapy. Specifically, D-dimer is known to be elevated during the acute phase of ischemic events due to fibrin turnover and the initiation of anticoagulation therapy, which could artificially increase its levels and obscure its role as a pre-existing susceptibility marker. This potential confounding effect highlights the need for caution in interpreting these biomarkers as independent risk factors for PFO-related stroke. To gain more accurate insights into the biological mechanisms at play, future studies should include a comparison of biomarker levels between the acute and subacute phases, as well as measurements taken before the ischemic event, ideally in a cohort with longitudinal follow-up. This conceptual issue is a limitation of our study because without longitudinal measurements or baseline data, it is difficult to distinguish whether the observed associations reflect pre-existing susceptibility, acute post-event effects, or hospital-related factors.

Elevated D-dimer in ischemic stroke may also reflect malignancy-associated hypercoagulability. Although patients with known active malignancy at the time of the index hospitalization were excluded, this retrospective study did not include standardized screening for occult malignancy; therefore, residual confounding from undiagnosed cancer cannot be fully excluded. Future prospective studies should incorporate systematic assessment of malignancy status when evaluating D-dimer–based prediction models in stroke populations.

The model’s performance in the independent test set, which achieved an area under the curve (AUC) of 0.990, may be overly optimistic given the modest sample size (*n* = 300) and the single-center retrospective design. While we acknowledge that the cohort in this study was pre-selected, including females who had already undergone PFO-specific imaging, the study was designed with a specific goal: to develop a model that prioritizes PFO testing in females already referred for evaluation. This cohort, enriched for patients with a higher likelihood of PFO, limits the generalizability of the model for early identification in a broader population.

The SHAP-based interpretation highlights estradiol and D-dimer as strong contributors to PFO prediction. In clinical settings, elevated levels of these biomarkers in a patient with cryptogenic stroke or TIA may help support earlier consideration of confirmatory PFO testing (e.g., contrast-enhanced TCD or TEE), particularly when resources are limited or when conventional risk scores are inconclusive. In this study, we did not evaluate a biomarker-driven diagnostic pathway or downstream clinical outcomes; therefore, any workflow integration should be considered hypothesis-generating. Nonetheless, our findings suggest that a biomarker-informed model could function as a clinical prioritization tool to identify patients who may warrant timely PFO-focused imaging, which should be tested prospectively before clinical implementation.

Current practice often relies on anatomical imaging after the ischemic event has occurred, typically using TEE, which is invasive, resource-dependent, and not readily available in all settings (23). Our model demonstrates that routinely collected clinical and laboratory parameters including age, estradiol, FSH, D-dimer, and LDL-C can achieve high discriminative performance for PFO status, with an AUC of 0.990 in the held-out test set. Importantly, this performance was achieved using logistic regression, a widely understood and easily implemented algorithm that retains interpretability without sacrificing predictive accuracy. By adding SHAP, we did not imply that LR lacks interpretability; rather, SHAP was used to provide patient-level explanations and to present feature contributions on the probability scale in a way that is easily visualized for clinicians. This is particularly relevant in stroke care, where clinical decisions regarding PFO evaluation and secondary prevention have long-term implications and should ideally be supported by transparent, evidence-based tools. The use of SHAP plots to visualize how specific features influence each prediction may enhance clinician trust in the model’s outputs and facilitate shared decision-making with patients. These findings further suggest that the proposed model could facilitate rapid identification of patients who may benefit from prioritized confirmatory PFO testing (e.g., contrast-enhanced TCD or TEE), especially in urgent or resource-limited settings where immediate access to TEE/TCD is not available. Elevated estradiol and D-dimer levels point toward endothelial activation or a prothrombotic state, which in the presence of a right-to-left shunt may increase the plausibility of paradoxical embolic mechanisms; however, our study did not evaluate causality or treatment outcomes. Accordingly, we avoid interpreting these biomarkers as strengthening closure indications, and instead view the biomarker–clinical approach as potentially supporting timely diagnostic prioritization and specialist evaluation. These findings suggest that the proposed model could facilitate rapid identification of patients who may benefit from prioritized confirmatory PFO imaging, especially in urgent or resource-limited settings where immediate access to TEE/TCD is not available. Rather than implying causality or directly informing closure decisions, elevated estradiol and D-dimer may indicate a prothrombotic milieu that is associated with PFO presence in this female cohort. Accordingly, the model should be viewed as a triage tool to support PFO detection among referred patients, not as a tool to determine whether a detected PFO is pathogenic or whether closure should be performed; decisions about causal attribution and any treatment strategy (including closure) require established clinical assessment (e.g., RoPE/PASCAL) and prospective outcome-focused studies.

While the present design primarily supports use in primary or resource-limited hospitals where ultrasound imaging may not be immediately available, we do not view the application scope as overly narrow. Rather, the model can serve as a front-line screening or triage tool, accelerating recognition of patients who warrant confirmatory imaging. Beyond this, the integration of endocrine and coagulation biomarkers may also provide mechanistic insights into the pathophysiology of PFO-related stroke. However, it is important to acknowledge that the cohort used in this study consists of females who have already been selected for and undergone PFO-specific imaging. As such, the model addresses a different clinical question “Among females already referred and imaged for PFO, who has PFO?” rather than “Which females with cryptogenic stroke/TIA should be referred for PFO evaluation?” Future research should explore prospective studies with unselected or minimally selected populations to evaluate the model’s ability to guide the initial referral for PFO evaluation in females with cryptogenic stroke/TIA.

Our study also responds to a broader need for individualized stroke prevention strategies in the context of cryptogenic events, especially among younger patients who often lack traditional risk factors. By integrating endocrine and coagulation markers with machine learning, our model moves toward a biologically informed framework for stroke risk profiling in PFO carriers. Moreover, the inclusion of LDL-C as a retained predictor despite its modest standalone association—suggests a possible contribution of lipid-related endothelial dysfunction to embolic susceptibility, warranting further mechanistic investigation (24). Notably, LDL-C showed a negative contribution in the multivariable model. One plausible interpretation is that lower LDL-C may reflect a lower burden of traditional atherosclerotic risk factors (or greater use of lipid-lowering therapy), aligning with the clinical phenotype captured by RoPE (i.e., fewer conventional vascular risk factors among patients with cryptogenic events). Because LDL-C in our model represents a conditional association after accounting for age, hormones, and coagulation markers, this finding should be interpreted cautiously and warrants confirmation in external cohorts with treatment data (e.g., statin use) and broader vascular risk profiling.

The mere presence of a PFO does not necessarily confirm its causal role in a cryptogenic ischemic event. To address this, we incorporated RoPE and PASCAL classifications in the present revision to estimate the probability of a causal relationship. However, our analyses in this study are focused on PFO detection, and we did not evaluate clinical outcomes or treatment effects; therefore, we do not draw conclusions regarding PFO closure. Our findings suggest that the biomarker-based model can complement established scores for characterizing patients who warrant further diagnostic evaluation, particularly in patients with “possible” PASCAL classifications, settings in which clinical decision-making can be challenging. By integrating biomarkers that indicate endothelial activation or a prothrombotic state with causal likelihood scores, this approach may refine patient selection for further imaging and subsequent specialist assessment, especially in borderline cases.

Several limitations should be noted. First, biomarkers were collected within 48 h of the index event; acute-phase responses, stress, inflammation, and treatments (including anticoagulation) may influence levels particularly D-dimer limiting causal interpretation of biomarker–PFO associations. Second, although patients with known active malignancy were excluded, this retrospective study did not include standardized screening for occult malignancy; therefore, residual confounding from undiagnosed cancer-related hypercoagulability cannot be fully excluded. Third, this was a retrospective, single-center study with a modest sample size and internal testing only; the high AUC observed in the held-out test set may partly reflect selection (patients already referred for PFO imaging) and center-specific characteristics, and requires external and prospective validation in multicenter cohorts before clinical implementation. Fourth, while contrast-enhanced TCD combined with contrast echocardiography is practical and sensitive for right-to-left shunt detection, it is not fully equivalent to TEE for definitive anatomic characterization and may miss very small PFOs, potentially introducing misclassification. Finally, RoPE/PASCAL estimate the causal likelihood that a detected PFO is pathogenic, whereas our model predicts the probability of PFO presence among patients with cryptogenic stroke/TIA who underwent PFO testing; these tools therefore address distinct clinical questions, and future work should evaluate how biomarker-based prediction can be integrated within established diagnostic pathways. In conclusion, this study demonstrates that a parsimonious, interpretable model incorporating age, estradiol, FSH, D-dimer, and LDL-C can accurately predict PFO status among females with cryptogenic ischemic stroke or TIA. Prospective, multicenter validation with standardized biomarker timing and broader clinical characterization is needed before clinical implementation.

## Data Availability

The raw data supporting the conclusions of this article will be made available by the authors, without undue reservation.
