# Supplementary material for: Hormonal and laboratory predictors of patent foramen ovale in cryptogenic ischemic events: a SHAP-enhanced logistic regression approach
Source: Front Neurol. 2026 Apr 17;17:1738335. doi: 10.3389/fneur.2026.1738335 (PMC13132740; doi:10.3389/fneur.2026.1738335)
Supplement: Supplementary file 1 [file Supplementary_file_1.DOCX]

### SHAP-Based Interpretation

To enhance the transparency and interpretability of the predictive model, SHAP were applied to the final logistic regression model. SHAP is a game-theoretic approach that attributes prediction outcomes to individual features, thereby enabling both global and local interpretability of machine learning models.

SHAP analysis was performed using the SHAP package (version 0.41.0) in Python and was conducted at three interpretability levels: (1) Global Feature Importance: The mean absolute SHAP values were calculated across all samples in the test set to quantify and rank the relative influence of each feature on the model’s predictions. (2) Feature Distribution and Directionality: A SHAP summary plot was used to visualize the distribution of SHAP values for each variable across the test population. Color gradients were applied to reflect the original feature values, providing insight into the directionality of each variable's effect. In addition, a SHAP dependence plot was created to explore the nonlinear relationship between estradiol levels and their contribution to PFO probability. (3) Individual-Level Explanations: SHAP force plots were generated for representative patients in the test set to illustrate how specific features contributed to increasing or decreasing the predicted risk of PFO on a case-by-case basis.

All SHAP visualizations were derived from the independent test dataset (n = 90) using the finalized logistic regression model, which had been trained on the full training set. These explainability analyses provided insight into how key variables such as estradiol, D-dimer, and age influenced the model’s decision-making process, thereby supporting its potential utility in clinical settings.

**SHAP-Based Interpretation Reveals Feature Contributions and Individual-Level Risk Patterns**

To enhance model interpretability, SHAP analysis was applied to the final logistic regression model. The summary plot revealed that estradiol (E2), D-dimer, and age were the top contributors to model output, with higher values generally associated with increased predicted risk of PFO (Figure SA). Ranking by mean absolute SHAP values further confirmed E2 as the most influential feature, followed by D-dimer and age (Figure SB). The SHAP dependence plot showed a nonlinear positive association between E2 levels and their corresponding SHAP contributions (Figure SC). Individual-level force plots illustrated representative prediction cases: one patient with low predicted probability (Figure SD), one with moderate probability (Figure 4E), and one with high probability of PFO (Figure SF), highlighting the directional impact of key features on each prediction.


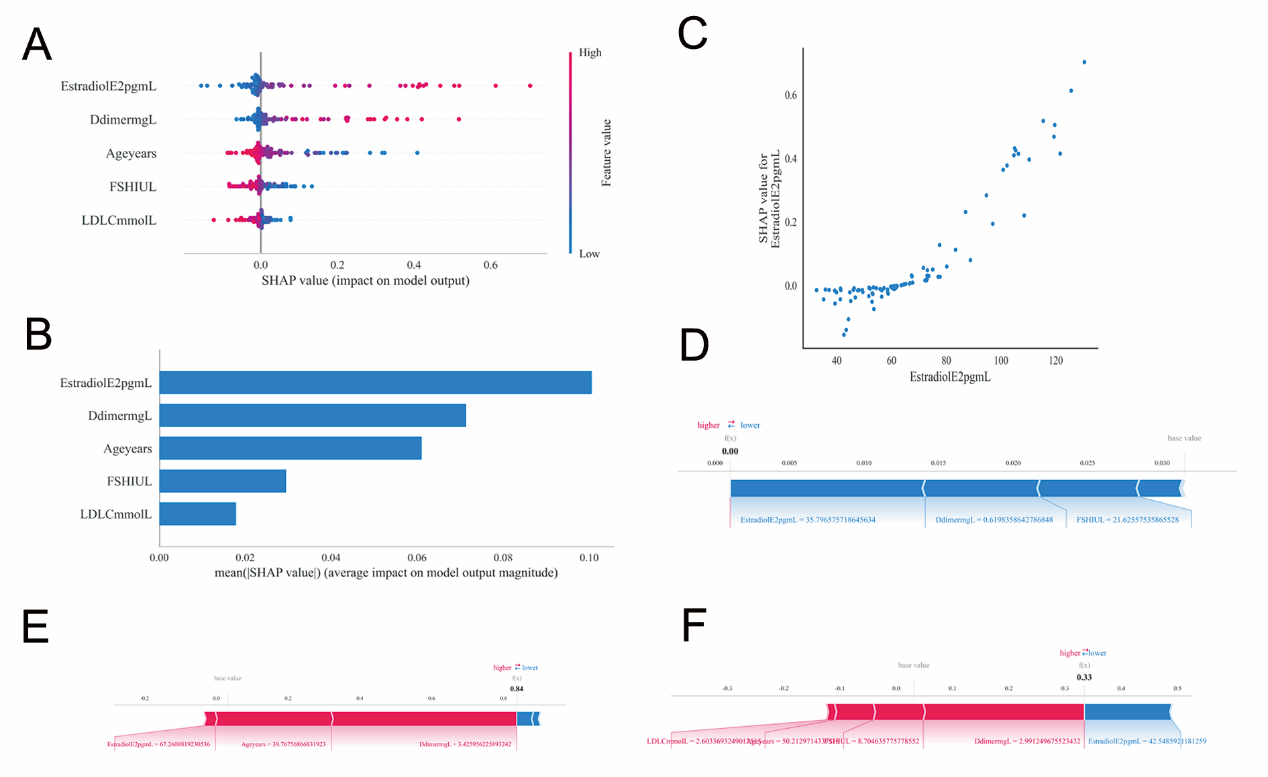


**Fig. S. SHAP-based interpretability analysis of the final logistic regression model.** (A) SHAP summary plot showing the distribution of SHAP values for each feature across all samples. Dot color represents the original feature value (red = high, blue = low). (B) Mean absolute SHAP values ranked by importance, indicating the average contribution of each variable to the model output. (C) SHAP dependence plot for estradiol (E2), showing the nonlinear relationship between its value and its SHAP contribution. (D–F) SHAP force plots for three representative individuals. Red and blue bars represent features that increase or decrease the predicted probability of PFO, respectively, with bar length reflecting contribution strength. Note: SHAP = Shapley additive explanations; PFO = patent foramen ovale; E2 = estradiol; FSH = follicle-stimulating hormone; LDL-C = low-density lipoprotein cholesterol.
