# Supplementary material for: Hormonal and laboratory predictors of patent foramen ovale in cryptogenic ischemic events: a SHAP-enhanced logistic regression approach
Source: Front Neurol. 2026 Apr 17;17:1738335. doi: 10.3389/fneur.2026.1738335 (PMC13132740; doi:10.3389/fneur.2026.1738335)
Supplement: Supplementary file 2 [file Table_1.docx]

**Table S1**. **Number and percentage of missing values for candidate variables included in model development**

| Variable | Missing (n) | Missing (%) |
| --- | --- | --- |
| Age | 3 | 1.0 |
| Sex | 3 | 1.0 |
| BMI | 3 | 1.0 |
| Smoking history | 3 | 1.0 |
| Pregnancy history | 3 | 1.0 |
| Menopausal status | 3 | 1.0 |
| Ischemic event type | 3 | 1.0 |
| Lesion location | 3 | 1.0 |
| First-ever event | 3 | 1.0 |
| Estradiol | 12 | 4.0 |
| Progesterone | 12 | 4.0 |
| Prolactin | 3 | 1.0 |
| FSH | 12 | 4.0 |
| LH | 12 | 4.0 |
| Testosterone | 3 | 1.0 |
| D-dimer | 3 | 1.0 |
| Fibrinogen | 3 | 1.0 |
| Prothrombin time | 3 | 1.0 |
| APTT | 3 | 1.0 |
| Platelet count | 3 | 1.0 |
| hs-cTnI | 3 | 1.0 |
| NT-proBNP | 3 | 1.0 |
| BUN | 3 | 1.0 |
| Serum creatinine | 3 | 1.0 |
| Total cholesterol | 3 | 1.0 |
| Triglycerides | 3 | 1.0 |
| HDL-C | 3 | 1.0 |
| LDL-C | 3 | 1.0 |
| Fasting plasma glucose | 3 | 1.0 |
| HbA1c | 3 | 1.0 |
| WBC | 3 | 1.0 |
| Neutrophil percentage | 3 | 1.0 |

Data are presented as the absolute number (n) and percentage (%) of missing values for each candidate variable. No variable exceeded 5% missingness. Hormonal indicators (estradiol, progesterone, FSH, and LH) had the highest missing rates (3–5%), while all other variables had <2% missing data. Missing values were handled using multiple imputation by chained equations (MICE), and results were consistent in complete-case analyses.

**Table S2. mRMR ranking and scores for LASSO-retained candidate predictors**

| **Rank** | **Feature** | **mRMR score** | **Selected (Yes/No)** |
| --- | --- | --- | --- |
| 1 | Estradiol (E2) | 0.186 | Yes |
| 2 | D-dimer | 0.173 | Yes |
| 3 | Age | 0.158 | Yes |
| 4 | LDL-C | 0.121 | Yes |
| 5 | FSH | 0.109 | Yes |
| 6 | Lesion location (cortical) | 0.083 | No |
| 7 | Ischemic event type (TIA) | 0.079 | No |
| 8 | BMI | 0.071 | No |
| 9 | Smoking history | 0.062 | No |
| 10 | HDL-C | 0.057 | No |
| 11 | HbA1c | 0.052 | No |
| 12 | White blood cell count | 0.049 | No |
| 13 | Neutrophil percentage | 0.046 | No |
| 14 | Total cholesterol | 0.043 | No |
| 15 | Triglycerides | 0.041 | No |
| 16 | Progesterone | 0.038 | No |
| 17 | Testosterone | 0.035 | No |
| 18 | Fibrinogen | 0.033 | No |
| 19 | Platelet count | 0.031 | No |
| 20 | PT | 0.028 | No |
| 21 | hs-cTnI | 0.025 | No |
| 22 | BUN | 0.023 | No |
| 23 | APTT | 0.020 | No |
| 24 | Serum creatinine | 0.018 | No |

Footnote: mRMR = maximum relevance minimum redundancy. Scores shown are placeholders for formatting only. Report the true mRMR scores (or criterion values) and ranking order from your software output in the final manuscript.

**Table S3. Coefficients of variables selected by LASSO regression at optimal lambda values**

| Variable | Coefficient at λ_min (λ = 0.008) | Coefficient at λ_1SE (λ = 0.026) |
| --- | --- | --- |
| Intercept | 32.627 | 12.345 |
| Smoking history | –0.032 | 0.000 |
| Pregnancy history | 0.070 | 0.000 |
| Postmenopausal status | –0.823 | 0.000 |
| Ischemic event type (TIA) | –0.299 | 0.097 |
| Lesion location (cortical) | 0.730 | 0.333 |
| Age (years) | –0.175 | –0.111 |
| BMI (kg/m²) | –0.115 | –0.037 |
| Estradiol (E2, pg/mL) | 0.064 | 0.045 |
| Progesterone (P, ng/mL) | 0.060 | 0.000 |
| Prolactin (PRL, ng/mL) | 0.064 | 0.000 |
| FSH (IU/L) | –0.068 | –0.022 |
| LH (IU/L) | 0.000 | 0.000 |
| Testosterone (T, ng/dL) | 0.002 | 0.000 |
| D-dimer (mg/L) | 1.667 | 1.061 |
| Fibrinogen (g/L) | –0.556 | –0.007 |
| Prothrombin time (PT, s) | –1.397 | –0.745 |
| APTT (s) | 0.000 | 0.000 |
| Platelet count (×10⁹/L) | –0.011 | –0.003 |
| hs-cTnI (pg/mL) | –0.081 | 0.000 |
| NT-proBNP (pg/mL) | 0.000 | 0.000 |
| BUN (mmol/L) | –0.126 | 0.000 |
| Serum creatinine (μmol/L) | 0.000 | 0.000 |
| Total cholesterol (TC, mmol/L) | –0.074 | 0.000 |
| Triglycerides (TG, mmol/L) | –0.241 | 0.000 |
| HDL-C (mmol/L) | 0.464 | 0.000 |
| LDL-C (mmol/L) | –0.993 | –0.452 |
| HbA1c (%) | –0.575 | –0.213 |
| White blood cell count (×10⁹/L) | 0.036 | 0.000 |
| Neutrophil percentage (%) | –0.012 | 0.000 |

Coefficients were estimated using 10-fold cross-validation at two regularization parameters: λ_min, corresponding to the minimum binomial deviance (λ = 0.008), and λ_1SE, corresponding to the most parsimonious model within one standard error (λ = 0.026). Variables with non-zero coefficients at either λ value were considered for subsequent mRMR-based refinement and classification modeling. Note: LASSO = least absolute shrinkage and selection operator; FSH = follicle-stimulating hormone; LH = luteinizing hormone; LDL-C = low-density lipoprotein cholesterol; HDL-C = high-density lipoprotein cholesterol; hs-cTnI = high-sensitivity cardiac troponin I; BUN = blood urea nitrogen.

**Table S4. Performance metrics of nine classifiers in the training cohort (n = 210)**

| Model | AUC (SD) | Cutoff (SD) | Accuracy (SD) | Sensitivity (SD) | Specificity (SD) | PPV (SD) | NPV (SD) | F1 Score (SD) | Kappa (SD) |
| --- | --- | --- | --- | --- | --- | --- | --- | --- | --- |
| XGBoost | 1.000 (0.000) | 0.806 (0.023) | 1.000 (0.000) | 1.000 (0.000) | 1.000 (0.000) | 1.000 (0.000) | 1.000 (0.000) | 1.000 (0.000) | 1.000 (0.000) |
| Logistic | 0.963 (0.004) | 0.190 (0.012) | 0.986 (0.018) | 0.893 (0.038) | 0.993 (0.009) | 0.685 (0.055) | 0.976 (0.016) | 0.779 (0.024) | 0.713 (0.035) |
| LightGBM | 1.000 (0.000) | 0.866 (0.017) | 1.000 (0.000) | 1.000 (0.000) | 1.000 (0.000) | 1.000 (0.000) | 1.000 (0.000) | 1.000 (0.000) | 1.000 (0.000) |
| Random Forest | 1.000 (0.000) | 0.525 (0.046) | 0.999 (0.002) | 1.000 (0.000) | 0.999 (0.004) | 0.995 (0.008) | 1.000 (0.000) | 0.997 (0.004) | 0.997 (0.005) |
| AdaBoost | 1.000 (0.000) | 0.502 (0.003) | 0.999 (0.002) | 1.000 (0.000) | 0.999 (0.002) | 0.995 (0.008) | 1.000 (0.000) | 0.997 (0.004) | 0.997 (0.005) |
| Decision Tree | 1.000 (0.000) | 1.000 (0.000) | 1.000 (0.000) | 1.000 (0.000) | 1.000 (0.000) | 1.000 (0.000) | 1.000 (0.000) | 1.000 (0.000) | 1.000 (0.000) |
| GBDT | 1.000 (0.000) | 0.624 (0.039) | 1.000 (0.000) | 1.000 (0.000) | 1.000 (0.000) | 1.000 (0.000) | 1.000 (0.000) | 1.000 (0.000) | 1.000 (0.000) |
| Gaussian NB | 0.957 (0.003) | 0.210 (0.044) | 0.877 (0.014) | 0.928 (0.025) | 0.864 (0.037) | 0.634 (0.071) | 0.980 (0.019) | 0.752 (0.024) | 0.674 (0.042) |
| Complement NB | 0.922 (0.005) | 0.290 (0.182) | 0.843 (0.017) | 0.907 (0.029) | 0.826 (0.028) | 0.569 (0.033) | 0.973 (0.018) | 0.698 (0.060) | 0.600 (0.053) |

Model discrimination, threshold, and classification performance were evaluated using 10-fold cross-validation. Most ensemble models demonstrated perfect in-sample performance, indicating potential overfitting. Logistic regression showed strong and balanced performance without overfitting. Note: AUC = area under the curve; SD = standard deviation; PPV = positive predictive value; NPV = negative predictive value; F1 = F1 harmonic mean of precision and recall; GBDT = gradient boosting decision tree; NB = naïve Bayes.

**Table S5. Performance metrics of nine classifiers in the held-out test set (n = 90)**

| Model | AUC (SD) | Cutoff (SD) | Accuracy (SD) | Sensitivity (SD) | Specificity (SD) | PPV (SD) | NPV (SD) | F1 Score (SD) | Kappa (SD) |
| --- | --- | --- | --- | --- | --- | --- | --- | --- | --- |
| XGBoost | 0.922 (0.027) | 0.806 (0.023) | 0.857 (0.047) | 0.467 (0.194) | 0.954 (0.044) | 0.770 (0.232) | 0.879 (0.041) | 0.552 (0.157) | 0.476 (0.179) |
| Logistic | 0.958 (0.033) | 0.180 (0.012) | 0.860 (0.059) | 0.867 (0.163) | 0.858 (0.070) | 0.622 (0.122) | 0.947 (0.051) | 0.714 (0.114) | 0.648 (0.148) |
| LightGBM | 0.921 (0.042) | 0.866 (0.017) | 0.863 (0.053) | 0.417 (0.281) | 0.975 (0.028) | – | 0.874 (0.055) | – | 0.442 (0.273) |
| Random Forest | 0.928 (0.038) | 0.525 (0.046) | 0.873 (0.025) | 0.633 (0.125) | 0.933 (0.028) | 0.711 (0.073) | 0.912 (0.027) | 0.662 (0.078) | 0.585 (0.092) |
| AdaBoost | 0.924 (0.047) | 0.502 (0.003) | 0.870 (0.067) | 0.617 (0.289) | 0.933 (0.062) | 0.716 (0.062) | 0.912 (0.034) | 0.626 (0.064) | 0.555 (0.261) |
| Decision Tree | 0.704 (0.120) | 1.000 (0.000) | 0.817 (0.065) | 0.517 (0.229) | 0.892 (0.053) | 0.554 (0.067) | 0.883 (0.049) | 0.519 (0.060) | 0.408 (0.230) |
| GBDT | 0.929 (0.033) | 0.624 (0.039) | 0.863 (0.050) | 0.550 (0.236) | 0.942 (0.046) | 0.735 (0.181) | 0.897 (0.051) | 0.597 (0.063) | 0.521 (0.187) |
| Gaussian NB | 0.947 (0.038) | 0.210 (0.044) | 0.867 (0.042) | 0.867 (0.163) | 0.867 (0.067) | 0.628 (0.077) | 0.948 (0.039) | 0.719 (0.053) | 0.636 (0.142) |
| Complement NB | 0.920 (0.041) | 0.290 (0.182) | 0.827 (0.053) | 0.867 (0.100) | 0.552 (0.060) | 0.569 (0.093) | 0.936 (0.029) | 0.671 (0.067) | 0.563 (0.119) |

Logistic regression demonstrated the most balanced performance, with the highest AUC, sensitivity, F1 score, and kappa. Most ensemble models prioritized specificity, resulting in reduced sensitivity. Decision Tree performed poorly across metrics. Note: AUC = area under the curve; SD = standard deviation; PPV = positive predictive value; NPV = negative predictive value; F1 = F1 harmonic mean of precision and recall; GBDT = gradient boosting decision tree; NB = naïve Bayes; “–” indicates undefined value due to class imbalance or missing calculation.

**Table S6A. Pairwise DeLong test P values comparing AUCs of nine classification models in the validation set**

| Comparison | XGBoost | Logistic | LightGBM | RF | AdaBoost | DT | GBDT | GNB | CNB |
| --- | --- | --- | --- | --- | --- | --- | --- | --- | --- |
| XGBoost | – | 0.158 | 0.972 | 0.972 | 0.793 | 0.008 | 0.846 | 0.387 | 0.979 |
| Logistic |  | – | 0.123 | 0.308 | 0.047 | 0.047 | 0.133 | 0.331 | 0.112 |
| LightGBM |  |  | – | 0.947 | 0.723 | 0.006 | 0.938 | 0.223 | 0.998 |
| Random Forest (RF) |  |  |  | – | 0.629 | 0.020 | 0.999 | 0.148 | 0.933 |
| AdaBoost |  |  |  |  | – | 0.017 | 0.610 | 0.058 | 0.709 |
| Decision Tree (DT) |  |  |  |  |  | – | 0.017 | 0.003 | 0.008 |
| GBDT |  |  |  |  |  |  | – | 0.113 | 0.924 |
| Gaussian NB (GNB) |  |  |  |  |  |  |  | – | 0.190 |
| Complement NB (CNB) |  |  |  |  |  |  |  |  | – |

Values indicate whether differences in discrimination performance are statistically significant. Logistic regression significantly outperformed Decision Tree and AdaBoost (P = 0.047), while other comparisons showed no statistically significant differences. Note: AUC = area under the curve; P values <0.05 are considered significant and shown in bold. RF = Random Forest; DT = Decision Tree; GBDT = gradient boosting decision tree; GNB = Gaussian naïve Bayes; CNB = complement naïve Bayes; “–” = not applicable (self-comparison).

**Table S6B. Pairwise DeLong test Z statistics comparing AUCs of nine classification models in the held-out test set set**

| Comparison | XGBoost | Logistic | LightGBM | RF | AdaBoost | DT | GBDT | GNB | CNB |
| --- | --- | --- | --- | --- | --- | --- | --- | --- | --- |
| XGBoost | – | –1.416 | 0.036 | 0.035 | 0.264 | 2.660 | 0.194 | –0.864 | 0.026 |
| Logistic |  | – | 1.539 | 1.021 | 1.987 | 1.990 | 1.508 | 0.974 | 1.589 |
| LightGBM |  |  | – | 0.066 | 0.353 | 2.776 | 0.078 | 1.215 | 0.002 |
| Random Forest (RF) |  |  |  | – | 0.483 | 2.326 | –0.001 | 1.447 | 0.083 |
| AdaBoost |  |  |  |  | – | 2.398 | 0.514 | 1.900 | 0.377 |
| Decision Tree (DT) |  |  |  |  |  | – | 2.398 | 2.930 | 2.652 |
| GBDT |  |  |  |  |  |  | – | 1.582 | 0.096 |
| Gaussian NB (GNB) |  |  |  |  |  |  |  | – | 1.312 |
| Complement NB (CNB) |  |  |  |  |  |  |  |  | – |

Positive values indicate that the model in the row outperformed the model in the column in terms of discrimination; values near zero reflect similar AUCs. Z ≥ ±1.96 corresponds to P < 0.05. Note: AUC = area under the curve; RF = Random Forest; DT = Decision Tree; GBDT = gradient boosting decision tree; GNB = Gaussian naïve Bayes; CNB = complement naïve Bayes; “–” = not applicable (self-comparison).

**Table S7. Summary of logistic regression model performance across the training set, 10-fold cross-validation (training set), and held-out test set**

| **Dataset** | **AUC (SD)** | **Cutoff (SD)** | **Accuracy (SD)** | **Sensitivity (SD)** | **Specificity (SD)** | **PPV (SD)** | **NPV (SD)** | **F1 Score (SD)** |
| --- | --- | --- | --- | --- | --- | --- | --- | --- |
| Training set (apparent performance; n = 210) | 0.948 (0.005) | 0.201 (0.058) | 0.862 (0.014) | 0.916 (0.028) | 0.849 (0.023) | 0.598 (0.032) | 0.977 (0.007) | 0.723 (0.018) |
| 10-fold cross-validation (within training set; n = 210) | 0.938 (0.043) | 0.201 (0.058) | 0.843 (0.074) | 0.855 (0.163) | 0.840 (0.079) | 0.599 (0.176) | 0.960 (0.046) | 0.689 (0.128) |
| Held-out test set (single split; n = 90) | 0.990 (-) | 0.286 (-) | 0.956 (-) | 0.895 (-) | 0.972 (-) | 0.895 (-) | 0.972 (-) | 0.895 (-) |

Results include area under the ROC curve (AUC), optimal probability cutoff, classification accuracy, sensitivity, specificity, positive predictive value (PPV), negative predictive value (NPV), and F1 score. Standard deviations (SD) are shown for the training set and 10-fold cross-validation results. “10-fold cross-validation” corresponds to internal validation performed exclusively within the training set; no external held-out test set was used. The held-out test set metrics reflect evaluation on a single 30% split (n = 90) and therefore do not have an associated SD. Note: AUC = area under the curve; PPV = positive predictive value; NPV = negative predictive value; SD = standard deviation; F1 = harmonic mean of precision and recall; PFO = patent foramen ovale.
